# Cell Replacement Therapy for Brain Repair: Recent Progress and Remaining Challenges for Treating Parkinson’s Disease and Cortical Injury

**DOI:** 10.3390/brainsci13121654

**Published:** 2023-11-29

**Authors:** Paul M. Harary, Dennis Jgamadze, Jaeha Kim, John A. Wolf, Hongjun Song, Guo-li Ming, D. Kacy Cullen, H. Isaac Chen

**Affiliations:** 1 Department of Neurosurgery, Perelman School of Medicine, University of Pennsylvania, Philadelphia, PA 19104, USA; pharary@stanford.edu (P.M.H.);; 2 Corporal Michael J. Crescenz Veterans Affairs Medical Center, Philadelphia, PA 19104, USA; 3 Department of Neuroscience and Mahoney Institute for Neurosciences, Perelman School of Medicine, University of Pennsylvania, Philadelphia, PA 19104, USA; 4 Institute for Regenerative Medicine, University of Pennsylvania, Philadelphia, PA 19104, USA; 5 Department of Cell and Developmental Biology, Perelman School of Medicine, University of Pennsylvania, Philadelphia, PA 19104, USA; 6 The Epigenetics Institute, Perelman School of Medicine, University of Pennsylvania, Philadelphia, PA 19104, USA; 7 Department of Psychiatry, Perelman School of Medicine, University of Pennsylvania, Philadelphia, PA 19104, USA; 8 Department of Bioengineering, School of Engineering and Applied Science, University of Pennsylvania, Philadelphia, PA 19104, USA

**Keywords:** neural replacement, brain organoids, tissue engineering, Parkinson’s disease

## Abstract

Neural transplantation represents a promising approach to repairing damaged brain circuitry. Cellular grafts have been shown to promote functional recovery through “bystander effects” and other indirect mechanisms. However, extensive brain lesions may require direct neuronal replacement to achieve meaningful restoration of function. While fetal cortical grafts have been shown to integrate with the host brain and appear to develop appropriate functional attributes, the significant ethical concerns and limited availability of this tissue severely hamper clinical translation. Induced pluripotent stem cell-derived cells and tissues represent a more readily scalable alternative. Significant progress has recently been made in developing protocols for generating a wide range of neural cell types in vitro. Here, we discuss recent progress in neural transplantation approaches for two conditions with distinct design needs: Parkinson’s disease and cortical injury. We discuss the current status and future application of injections of dopaminergic cells for the treatment of Parkinson’s disease as well as the use of structured grafts such as brain organoids for cortical repair.

## 1. Introduction

In December 1999, on the eve of the 21st century, Professors Richard Winn and Matthew Howard published an opinion piece in *The Lancet* with predictions for what would transpire in the next 100 years in neurosurgery [1]. Their central thesis described a shift from the mechanical era to a biological era of neurosurgery, with therapies increasingly focused on interventions at the cellular level. In many ways, this idea has proven to be extremely prescient. In recent years, research into neural repair via cellular transplantation has grown to include increasingly sophisticated cell constructs and methods, including the use of self-organizing brain organoids. 

Cell therapy is thought to mediate neural repair through two categories of mechanisms. The first category relies on activation of “bystander effects” that facilitate endogenous regeneration mechanisms and modulate immune responses. The second category is the replacement of damaged tissue to directly restore function [2,3]. In this paradigm, engrafted neurons incorporate into host neuronal networks to supplement or restore brain function and cerebral processing capacity. In cases of moderate to severe brain damage, bystander effects and endogenous repair may be insufficient to restore function. Neural substrate expansion to directly replace damaged cells is the natural alternative. 

An early strategy for neural replacement therapy involved the transplantation of fetal neural tissue into a damaged brain region. In 1979, a pair of landmark studies were published describing a significant reduction in motor abnormalities in parkinsonian rats following transplantation of mesencephalic tissue, which contains a high proportion of dopaminergic neuroblasts, into the caudate nucleus [4,5]. These successes led to two double-blind clinical trials in the late 1990s of intrastriatal implantation of fetal mesencephalic tissue in Parkinson’s disease patients [6,7]. Similar fetal tissue-based approaches were concurrently explored for cortical repair, with rodent fetal cortex transplanted into injury cavities in the cortex of adult rodents [8,9,10]. These fetal cortical grafts were shown to integrate with host brain and appeared to develop appropriate functional attributes [4,11,12]. These fetal grafts also formed targeted long-distance connections within the host brain as well as participated in limb movements [12,13]. However, the significant ethical concerns and limited availability of fetal tissue have limited their clinical translation. 

Induced pluripotent stem cell (iPSC)-derived cultures represent a more readily translatable alternative [14,15,16], with iPSC-derived cell systems spanning from monolayer cortical cultures to region-specific brain organoids. Great progress has been made in developing protocols for generating a wide range of neural cell types in vitro. In addition, autologous iPSCs have been used to generate patient-matched grafts, which enable transplantation strategies that dispense with the need for immunosuppression [17]. Transplanted iPSC-derived neurons have been shown to behave similarly to fetal neural tissue in preclinical models in several key respects, including long-distance axonal outgrowth [18,19,20] and their electrophysiological and neurochemical properties [15,21]. 

Overall funding for stem cell research in the United States has gradually increased over the past 25 years, greatly buoyed by private and individual state-level investments [22,23,24]. Total stem cell research funding by the National Institutes for Health consistently increased from fiscal year 2013 to fiscal year 2024, rising from 1.273 billion to 2.365 billion dollars [25]. In addition, state governments, most notably California, have made significant commitments to stem cell and regenerative medicine research. The California Institute for Regenerative Medicine has committed 3 billion dollars to these efforts, including several large projects involving transplantation-based repair [26].

In aggregate, these developments have stimulated significant interest in applying neural replacement therapy to a range of neurological conditions. To optimize the success of these therapies, it is essential to consider the context of the disease process for which neural replacement therapy is being applied, especially with respect to the design parameters of the ideal graft. Neural replacement therapy is far from “one-size-fits-all”. To illustrate this point, we discuss recent progress in neural transplantation approaches for two specific conditions with distinct design needs: Parkinson’s disease (PD) and cortical injury. We consider treatment approaches for PD directed at dopamine replenishment, namely injections of dissociated dopamine progenitor cells, as well as strategies aimed at more extensive reconstitution of the nigrostriatal pathway. By comparison, strategies for cortical repair may require more highly structured substrates that reflect the organization of cortex. To this end, we review recent developments for engrafted brain organoids as well as remaining challenges for clinical translation. 

## 2. Neural Repair Approaches Should Reflect Disease-Specific Needs

**Figure 1:**
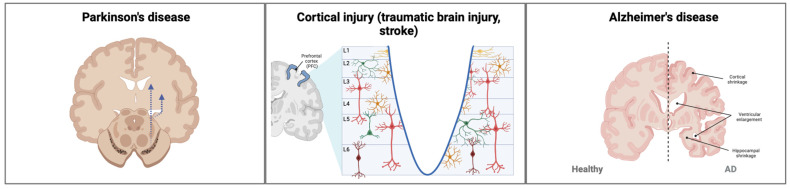
**Design needs of neural replacement approaches for different neurological conditions.** Many classical symptoms of Parkinson’s disease are caused by a decrease in dopaminergic innervation of the striatum due to loss of dopaminergic cells in the substantia nigra pars compacta. Dashed arrows represent the nigrostriatal pathway. By comparison, the cerebral cortex has an elaborate cytoarchitecture comprising six layers, which is disrupted in conditions such as traumatic brain injury and stroke. L1 represents the most superficial cortical layer, while L6 represents the deepest layer. In Alzheimer’s disease (AD) the hippocampus is significantly affected, often accompanied by ventricular enlargement and cortical shrinkage. Therefore, neural replacement strategies for each of these conditions should reflect the structure of the damaged tissue.

Neurological disorders encompass a wide range of pathologies and neuroanatomical structures. Cell therapies should be tailored to restore function based on an understanding of the underlying neuroanatomy and disease mechanisms. While significant progress has recently been made towards developing therapies for many neurological conditions, PD and cortical injury represent a particularly interesting comparison due to the contrasting design needs of a neural replacement therapy for each of these diseases. Parkinson’s disease primarily affects a single cell type, dopaminergic neurons in the substantia nigra (SN) of the midbrain. On the other hand, cortical injury, such as traumatic brain injury, stroke, and surgical resection of lesions, involves disruption of a highly organized horizontal and vertical structure formed by many cell types (Figure 1). Therefore, neural replacement approaches for these two conditions have disparate needs that reflect these differences. Cell therapies for other conditions will require tailoring the repair substrates to the structure of the relevant affected brain regions.

Neural repair for PD has long been focused on the replacement of dopamine in the basal ganglia [4,5,6,7]. The fundamental pathology of PD is the loss of dopaminergic neurons in the substantia nigra pars compacta (SNpc), which projects to the striatum [27,28]. The creation of clinically translatable dopaminergic neurons has been an iterative process, and it has recently yielded promising methodologies for generating the desired cells [20,29]. As therapeutic strategies move forward, several key design needs for PD bear careful consideration. First, the SNpc is primarily comprised of densely clustered DA cell nuclei and lacks extensive laminar architecture [30]. This anatomy supports injection of dissociated cells as a viable treatment for PD, whereas dissociated cells may be unsuitable for repair of more highly structured brain regions. Second, the location for injections, either the SN or striatum, is an important parameter. Intrastriatal transplantation has the advantage of delivering DA near to its target structure, with transplanted DA cells requiring only short-distance axonal outgrowth to synapse with host striatal neurons [31,32,33]. However, this ectopic placement prevents engrafted cells from receiving appropriate afferent inputs and participating in the basal ganglia feedback circuit. By comparison, intranigral transplantation may represent a more effective approach for true reconstruction of the nigrostriatal pathway [34]. The challenge with this approach is the need for intranigral grafts to send long-distance axons to the striatum [35], an obstacle that may limit the degree of DA replenishment compared to intrastriatal grafts. Therefore, while recreating the nigrostriatal pathway may represent the most complete repair of degenerated host circuitry, it bears consideration whether the benefits of normal feedback signaling to the SN are outweighed by the added technical requirements of intranigral grafting.

In contrast, effective neural replacement therapy for cortical injury will likely require the use of a neural substrate that reproduces the structural and functional complexity of the cerebral cortex. The high degree of vertical and horizontal structure within the cortex gives rise to the cortical circuitry that mediates information processing and supports a wide range of cognitive functions [36]. The laminar organization of neocortex, which is comprised of six distinct cellular layers, enables specific patterns of connectivity, with each layer having distinct sources of afferents and efferents [37]. This pattern of information flow through cortical layers gives rise to the canonical cortical “microcircuit”, which performs processing that is central to cortical function. Functionally similar cells are also radially aligned to form cortical columns, with variations within patterns of vertical and horizontal connectivity contributing to differences in circuitry across different cortical regions [38]. Lastly, interneurons within these columns form extensive connections to modulate network activity [39,40,41]. Given these important features, a cell product designed for cortical repair should possess sufficient structure to support sophisticated function. 

In the remainder of this review, we will discuss recent progress in the development of neural replacement strategies for PD and cortical repair. We will consider how these approaches address the design needs of cell therapies for each condition, as well as areas for further investigation. 

## 3. Restoring the Nigrostriatal Pathway in Parkinson’s Disease

Parkinson’s disease is a neurodegenerative disorder that is pathologically characterized by the progressive loss of dopaminergic neurons projecting to the striatum from the SN of the midbrain. The lack of dopaminergic innervation results in disruption of the direct and indirect pathways of the basal ganglia, which causes the tremor and rigidity that are the clinical hallmarks of PD. While current treatments such as levodopa and deep brain stimulation can mitigate PD symptoms, they do not address the underlying death of DA neurons that causes motor impairment. Cell transplantation therapy that directly replaces lost DA neurons with exogenous cells has therefore long been the focus of intense research. Approaches that have been investigated for cell replacement in PD fall into the general categories of (1) fetal mesencephalic tissue, (2) stem cell-derived DA neurons, and (3) tissue-engineered constructs that mimic the nigrostriatal pathway

**a.**  **Transplantation of fetal mesencephalic tissue demonstrates proof-of-principle for neural replacement**

Early cell replacement efforts for PD demonstrated the high therapeutic potential of neural replacement therapy but also revealed several obstacles. In the 1980s, a series of pre-clinical transplantation studies was performed using DA progenitors from mid-trimester rat fetuses, guided by the premise that reinnervation of the denervated striatum would ameliorate symptoms of PD [4,42]. The authors observed recovery of motor function in transplanted animals, with the degree of improvement correlated with the degree of reinnervation of the host striatum by engrafted cells. The strength of these results, supported by additional experiments producing similar results when human fetal DA cells were transplanted into parkinsonian rats [43], led to the first clinical trial in 1987 by Madrazo and colleagues in Mexico [44]. This trial was shortly followed by the initiation of similar trials in the United States [45], England [46], Spain [47], France [48], and Belgium [49]. The results of this wave of early trials were heterogeneous, with significant benefits observed in some patients [50,51,52] and modest to no benefit in others [53,54]. In addition, wide discussion took place surrounding the significant ethical issues associated with fetal tissue research [55]. Notably, US federal policy prohibited the use of National Institutes of Health funding to support research into transplantation of human fetal tissue until it was overturned by the Clinton administration in 1993 [56]. While fetal mesencephalic tissue ultimately proved to be unsuitable for broad clinical use due to ethical and logistical concerns, these early studies created a foundation upon which subsequent approaches were built. Therefore, it is valuable to consider the successes and challenges of these initial trials of fetal mesencephalic tissue transplantation in detail and how they informed subsequent efforts. 

The degree of efficacy in clinical trials of fetal mesencephalic tissue transplantation was highly variable. This heterogeneity of clinical benefit was observed both between studies as well as between patients within an individual study [6,7,57,58]. While some cases were observed to have increases in striatal ^18^F-Dopa uptake at the transplanted site and moderate improvements in motor symptoms, other patients had limited graft survival or developed graft-induced side effects [6,7]. Several potentially causal factors were suggested, including problems with patient selection, immunosuppressive protocols, surgical approach, and trial design [6,7,58]. For example, older patients may have had more advanced disease and been non-responsive to levodopa before the start of these clinical trials [59]. In addition, this group of patients may have had a lower degree of neural plasticity as well as less diffuse PD pathology [6]. Furthermore, neuroinflammation is known to increase with age, such that older patients may have suffered from an increased post-transplantation immune response with subsequent reduced survival of engrafted cells [60]. Overall, these clinical trials suggested that DA cell replacement had the potential to reduce disease progression, but the underlying biology was not fully understood. Closer examination of trial results is therefore highly relevant in considering future therapies.

These initial fetal tissue transplantation clinical trials established proof-of-principle for long-term graft survival and striatal reinnervation, despite providing inconsistent clinical benefits. Post-mortem findings from 18 months [61] and 4 to 16 years post-transplantation [7,28,62] revealed extensive graft survival and striatal innervation. More recently, a post-mortem study reported graft survival in a single patient 24 years post-transplantation, representing the longest post-grafting interval reported to date [63]. Graft survival was also confirmed using ^18^F-dopa PET in clinical trials [6,64]. Collectively, these results indicate that long-term graft survival is clearly possible, although clinical improvements were variable. Notably, post-mortem immunohistochemical analysis showed microglial infiltrates in grafts for four patients, suggesting a sustained immune response [7]. Interestingly, strong graft survival and reinnervation of the striatum were not consistently correlated with clinical benefits, particularly in patients who were older at the time of transplantation [7,53,54]. It has been proposed that this may be related to progressive DA denervation in areas outside the graft-innervated region, such that patients with more advanced PD did not experience benefits [65]. Consequently, transplantation approaches may require implantation sites to be tailored to individual patient pathology to optimize functional outcomes. 

The potential for graft-induced side effects, primarily dyskinesias, was a key concern of the early clinical trials. In a 2001 double-blind study led by Curt Freed and colleagues, 5 out of 33 patients receiving a transplant developed dyskinesias [6]. Olanow and colleagues later reported an even higher rate of dyskinesias (13 of 23 patients), including three patients with dyskinesias that were disabling and required additional surgical intervention [7]. Several mechanisms have been suggested to underlie these graft-induced dyskinesias, including graft contamination by serotonergic neurons [66]. This hypothesis is supported by a significant attenuation of dyskinesias following administration of a 5-HT1A receptor agonist, busiprone, which reduces the activity of serotonergic cells [66,67]. The 5-HT1A autoreceptor, an inhibitory G-protein coupled receptor, is known to be highly expressed on serotonergic neurons, where it functions to hyperpolarize cells and decrease serotonin release [68]. Alternatively, dyskinesias may arise due to unequal distribution of injected cells, leading to “hot spots” of DA neurons [69,70]. Lastly, it is possible that dyskinesias are the result of inadequate feedback signaling to the intrastriatal DA cell grafts, resulting in uncontrolled levels of DA in the striatum. 

Despite heterogeneous outcomes, the strong graft survival and significant clinical benefit to some patients in these early fetal mesencephalic tissue transplantation trials catalyzed research into neural replacement strategies for treatment of PD. Neural stem cells (NSCs) were viewed as a potential alternative cell source, which could be injected into the nigrostriatal pathway and receive cues from the host microenvironment to differentiate towards a DA cell phenotype. Several early studies were performed based on this hypothesis, demonstrating promising functional and behavioral recovery in parkinsonian rodents transplanted with NSCs [71,72]. However, subsequent preclinical studies strongly suggested that the benefits of NSC grafts were mediated by bystander effects rather than cell replacement [73,74,75,76]. The long-term outlook of this approach remains to be determined. The strategy of stem cell-derived DA neurons has largely overtaken NSCs as a more effective strategy for true cell replacement in PD.

**b.**  **Stem cell-derived dopaminergic cells as a translatable alternative to fetal tissue**

Stem cell differentiation has been regarded as one of the most promising means to produce human DA cells for transplantation-based PD therapies. In this approach, in vitro culture conditions are used to pattern human stem cells to produce DA progenitor cells, which can then be isolated for transplantation. This approach bypasses both the ethical issues associated with fetal grafts as well as the low DA cell yield observed in NSC grafts. In 2011, the Studer lab published a protocol for efficient generation of midbrain DA neurons from iPSCs [77]. This method pairs floor plate induction with dual SMAD inhibition to reliably produce tyrosine hydroxylase-positive DA neurons. However, the reliance on animal-derived factors, namely KnockOut Serum Replacement (KOSR), posed a meaningful barrier to translation. When differentiation was attempted using synthetic alternatives to KOSR, the quality of resulting DA neurons was found to be significantly decreased. This problem was recently resolved by the Studer group in 2021 [29], a full decade after the publication of their initial floor plate protocol. Using bi-phasic WNT activation, DA neurons can now be consistently derived from iPSCs without introduction of contaminants from animal-derived reagents. 

This differentiation protocol was then used to perform preclinical studies using hESC-derived cryopreserved DA neurons [20]. Cryopreservation enables the delivery of a centrally generated cell product to different clinical locations, which would not be possible with fresh cells. The Tabar and Studer labs therefore tested the intrastriatal transplantation of cryopreserved hESC-derived DA cells, a final cell product labeled as MSK-DA01, into a parkinsonian rat model [20]. At 8 months post-transplantation, transplanted rats were found to have improved motor skills compared to animals injected with vehicle based on amphetamine-induced rotational testing. Transplanted animals were shown to have significantly fewer amphetamine-induced rotations per minute. The efficacy of the treatment was further supported by histological data showing axonal outgrowth of the engrafted cells. These findings set the stage for clinical trials, which began in May 2021. 

The Phase I safety study of MSK-DA01, an allogeneic cell product derived entirely from the WA09 hESC line, was conducted by BlueRock Therapeutics and Bayer. The study enrolled 12 patients who had been diagnosed with PD between 5 and 15 years before the start of the trial and still demonstrated response to levodopa [78]. Surgical transplantation of MSK-DA01 cells was performed to the post-commissural putamen bilaterally at one of two different doses: 5 patients received 0.9 million cells per putamen, and 7 patients received 2.7 million cells per putamen. Transplantation was followed by 1 year of immunosuppression. Recently, results were released indicating that the study met safety and tolerability objectives with no serious adverse events directly related to introduction of the DA cells [79]. Furthermore, initial findings indicated an improvement in motor symptoms. The Hauser Diary, a measurement of time patients spend symptomatic, indicated an improvement in both dosage groups, with the higher dose producing the most improvement [79,80,81]. Following these results, a Phase II study is being initiated with planned enrollment beginning in the first half of 2024 [79].

Other notable clinical trials of stereotactic injection of human pluripotent stem cell (hPSC)-derived DA progenitor cells have also been conducted in parallel (Table 1). Studies conducted by Kyoto University, Weill Cornell/Massachusetts General Hospital, and upcoming Aspen trials are all notable for their use of true autografts derived from patient-derived iPS cells. A set of clinical trials was initiated in Japan in 2018 (JMA-IIA00384, UMIN00003356) as a collaboration between the Center for iPS Cell Research and Application (*CiRA*), Kyoto Hospital, and Astellas Pharmaceuticals. These studies used similar inclusion criteria to the more recent BlueRock/Bayer trial, with seven patients receiving a single dose of 2.4 million iPSC-derived DA neurons in August 2018 [82]. A single patient also received implantation of DA progenitors derived from autologous iPSCs through a collaboration between Weill Cornell Medical Center and Massachusetts General Hospital [83,84]. In the Weill Cornell/Massachusetts General Hospital trial, stereotactic injection delivered cells into the left hemisphere of the putamen, with a second injection into the right hemisphere 6 months later. Stabilization or improvement of clinical symptoms was observed 18 to 24 months after DA cell injection, and no adverse events were reported. Collectively, these results are highly encouraging for the therapeutic potential of DA cell replacement for PD. 

There remain some outstanding questions and obstacles revealed by these trials, primarily related to optimal surgery delivery techniques, long-term outcomes, and scalability of approach. The surgical method used for graft implantation is critical for optimizing both the survival and distribution of injected cells. Schweitzer and colleagues developed a “columnar” injection protocol, wherein DA cells were deposited at a constant rate along a designated segment of the track created by the surgical cannula [83]. By using the cannula track itself and injecting the cell suspension at a constant rate while the cannula was removed, a uniform density of cells may be seeded with a greater contact surface between the graft and the adjacent host tissue. Further optimization of surgical techniques will likely lead to greater improvements in graft outcomes. 

The initial inflammatory response to the cell transplantation procedure is another key factor in determining survival of engrafted DA cells. A recent study highlighted the role of acute neuroinflammation in guiding the differential survival of tyrosine hydroxylase(TH)-positive and TH-negative engrafted cells [60]. Park and colleagues performed transplantation of DA progenitor cells into large lesions in rodent striatum, both with and without co-injection of autologous regulatory T-cells. Co-injection of regulatory T-cells resulted in a significant increase in survival of engrafted TH-positive cells at both two weeks and twenty weeks post-transplantation. In addition, co-injection with regulatory T-cells produced larger improvement in amphetamine-induced and apomorphine-induced rotation tests than DA progenitor cells alone. Overall, this suggests that immune cells clearly have a role to play in neural repair strategies. 

The long-term efficacy and side effects of DA cell therapy are also not yet fully understood. While case reports have described long-term outcomes of a small number of patients [57,63], more systematic studies are needed to make reliable inferences. The final cell type composition of DA cell products must be further studied over longer differentiation periods, given concerns associated with serotonergic cells possibly giving rise to dyskinesias. Additionally, the question of whether autologous grafts have genetic predispositions to PD pathologies over the long term remains unanswered. Furthermore, it may be the case that transplanted cells will become “infected” by pathologic alpha-synuclein from diseased native cells. Postmortem studies from trials of fetal tissue transplantation revealed a gradual accumulation of alpha-synuclein in graft cells [62,63], although data were insufficient to determine if there was any clear functional impact of this pathology.

The overall scalability of iPSC-derived DA progenitor cells remains unclear. While these cell products can theoretically be produced at large scale to clinical standards, the efficiency of the production process and necessary quality control testing have yet to be fully tested on a commercial scale. Processes such as cryopreservation of DA cells [20] may allow for distribution to various clinical sites. However, the efficacy of this approach must be assessed when more broadly implemented. Overall, it will be essential to maintain best practices as the manufacture of hPSC-derived DA cells is expanded. Results from a Phase III trial may provide further insight into these issues.

**c.**  **Tissue-engineered constructs to reconstruct the nigrostriatal pathway**

Tissue-engineered nigrostriatal pathway (TE-NSP) technology represents an alternative approach to cell therapy for PD that has the potential to not only replace decreased endogenous dopamine but also reconstruct the long-distance axonal connections that comprise the nigrostriatal pathway. While injections of dissociated dopaminergic cells may form grafts that effectively increase dopaminergic signaling in the striatum, they are very unlikely to rebuild degenerated nigrostriatal circuitry. Consequently, the DA cells may be unable to respond to feedback signaling that normally regulates the substantia nigra, and the appropriate timing of dopaminergic signaling in the striatum may not be restored [6,74,88]. This lack of feedback regulation may limit the effectiveness of DA cell therapy and contribute to increased risk of side effects such as dyskinesias.

**Figure 2:**
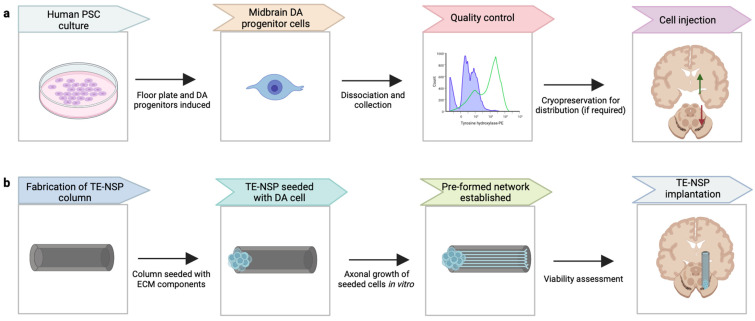
**Workflow for iPSC-derived dopaminergic progenitor cell treatment for Parkinson’s disease: Injection of dissociated cells and implantation of engineered microcolumns**. (**a**) A representation of the generation of midbrain dopaminergic (DA) progenitor cells by human pluripotent stem cell (PSC) culture, followed by quality control and cell intrastriatal (green arrow) or intranigral (red arrow) injection. (**b**) Tissue-engineered nigrostriatal pathway (TE-NSP) technology may be used to deliver DA cells to more specifically reconstitute nigrostriatal circuitry.

TE-NSPs, comprising a miniature tubular hydrogel seeded with dopaminergic neurons and extracellular matrix components [89,90,91,92,93], may allow for more effective restoration of the motor control feedback circuit between the SN and striatum (Figure 2). While TE-NSPs constructs typically have a diameter of only a few hundred microns, they can support long axonal growth. For example, a recent study demonstrated 9 mm of axonal after 28 days in vitro [94]. The seeding of TE-NSPs with neural aggregates rather than dissociated neurons produced a 10-fold increase in the rate of axonal outgrowth [94]. Importantly, TE-NSPs could be stereotactically injected without disruption of axonal projections within the column [34]. In a rat parkinsonian model, TE-NSP neurons survived in the SNpc, and axons extended to integrate within the striatum [95]. Also, TE-NSPs fabricated using hPSC-derived DA neurons were grown to scaled-up dimensions, comprising 200,000 DA neurons with axonal projections of >2 cm [93]. Seeding of columns with hPSC-derived DA cells and subsequent injection may therefore more directly address the design needs of neural replacement therapy for PD. TE-NSPs may also be used to deliver brain organoids with associated axon tracts [96], which contain multiple neural cell types and more extensive cytoarchitecture. A similar approach may be applied using midbrain organoids. Translation of this next-generation strategy for PD treatment is currently being spearheaded by Innervace, Inc. (New York, NY, USA).

There remain several key aspects of TE-NSP technology that need to be optimized for the clinical setting. The length of axonal outgrowth must be further increased to address the greater length of the nigrostriatal pathway in humans relative to animal models. In addition, survival of engrafted cells and maintenance of axonal integrity requires investigation in the context of a larger brain, with long-term follow-up. Finally, there are important surgical considerations associated with implanting TE-NSPs, which requires a different approach than the established procedure for injection of dissociated cells. While scaling up the strategy used for TE-NSP implantation into animal models [34] may represent a solution, long-term safety and efficacy must be verified for clinical translation. 

## 4. Structured Grafts for Cortical Repair

Repair of the cerebral cortex represents an area of great unmet clinical need, with no options currently available for functional replacement of lost tissue in conditions such as traumatic brain injury, stroke, and surgical resection of lesions. We propose that structured neural grafts that more closely reproduce normal cortical architecture are the optimal repair substrate for damaged cerebral cortex. The laminar architecture of cortex, described earlier, plays a key role in visual processing and has been shown to be highly preserved across other cortical areas [97,98]. Cortical layers connect to form a canonical microcircuit, with information flowing from the thalamus through the cortical laminae and then back to subcortical targets. In addition, layers II/III and V form horizontal, intralaminar connections. This structural arrangement, if present in an engineered tissue in vitro and preserved following transplantation, could result in structured grafts that more efficiently integrate with the host cortex and augment its computational capacity. Overall, the reproduction of tissue architecture may be one of the most notable advantages of using 3D relative to 2D cell systems as neural grafts for cortical repair.

**a.**  **Neural replacement as a treatment for cortical injury**

Investigation into transplantation-based cortical repair followed a similar trajectory to that discussed for PD. Pioneering experiments were performed using insertion of fetal cortical grafts into aspiration injury cavities in adult rats, with the authors reporting functional integration with host cortex at up to 10 months post-transplantation [11]. In another notable study, fetal cortical cells were inserted into adult rats following a fluid percussion injury, with observation of subsequent improvements of motor and cognitive function [99]. Further studies were performed using intracranial bone marrow transplantation [100] as well as NSCs derived from multipotent cell lines [101], with both approaches demonstrating functional improvement in small animal models of traumatic brain injury. Notably, however, the functional benefit associated with cell transplantation in these studies is likely to have been mediated by bystander effects. By comparison, seminal work by the Kokaia group showed that transplanted rat NSCs formed specific corticothalamic and contralateral hippocampal connections and received functional synaptic inputs from neighboring host neurons [102]. This evidence of functional integration of engrafted cells with host circuitry provided important support for neural replacement as a potential strategy for cortical repair. 

The development of more robust cortical neuron differentiation protocols enabled investigation into the use of hPSC-derived neurons to rebuild cortical circuits [103,104]. The Vanderhaeghen group transplanted hPSC-derived cortical neurons into neonatal mice, reporting integration and up to 9-month survival of engrafted cells [104]. Later, Vanderhaeghen and colleagues transplanted mouse ESC-derived neurons of visual cortex identity into an injury cavity in mouse visual cortex, observing formation of long-range reciprocal connections with the surrounding intact host circuitry [105]. In a subsequent study, intraventricular injection of hPSC-derived cortical neurons resulted in the integration of human neurons with mouse cortex and the adoption of orientation selectivity [106]. Furthermore, these studies demonstrated responses of engrafted neurons to visual sensory stimulation of host animals. Separately, the Kokaia group transplanted hPSC-derived cortical neurons into stroke-damaged rat cortex, resulting in the formation of efferent connections between engrafted cells and host cortical and subcortical areas [21]. In an additional set of experiments, engrafted hPSC-derived neurons were shown to receive inputs from host afferents and respond to sensory stimuli [107]. Collectively, these studies provide support for the ability of hPSC-derived neurons to efficiently integrate with host cortex in the setting of both intact and injured brain. 

Clinical trials of cell-based therapies for cortical injury remain ongoing, most notably for stroke [108]. Initial results have demonstrated that transplantation of bone marrow stem cells resulted in improvement on multiple stroke scales in patients with chronic stroke [109]. Notably, the study authors proposed that functional restoration observed in these trials was likely driven by “bystander effect”, such as trophic support provided by secreted factors, rather than true circuit reconstruction. While such an approach may be suitable for certain injuries, it is likely inadequate to address cases of more extensive tissue loss with less intact circuitry. Extensive integration of engrafted cells in the setting of large cortical injury has only been demonstrated using tissue-based grafts [11,12] rather than injection of dissociated cells. Therefore, structured grafts may be more suitable for direct neural replacement for treatment of significant cortical tissue loss.

**b.**  **Organoids as an alternative form of structured cortical graft**

While fetal cortical grafts may be ideal to use, their translation potential is limited. Brain organoids, which are self-organizing neural tissues generated from hPSCs, may be a viable alternative to fetal tissue. Forebrain organoids are produced using directed differentiation protocols and recapitulate several key features of human embryonic cortical development. Most notably, forebrain organoids have been shown to contain laminar structure containing upper- and lower-layer neurons [110,111] as well as outer radial glial cells [112,113]. Overall, organoids represent the lab-grown brain tissue that has by far the greatest degree of cortical architecture. Therefore, organoids are a strong candidate for use as a neural repair substrate.

**Figure 3:**
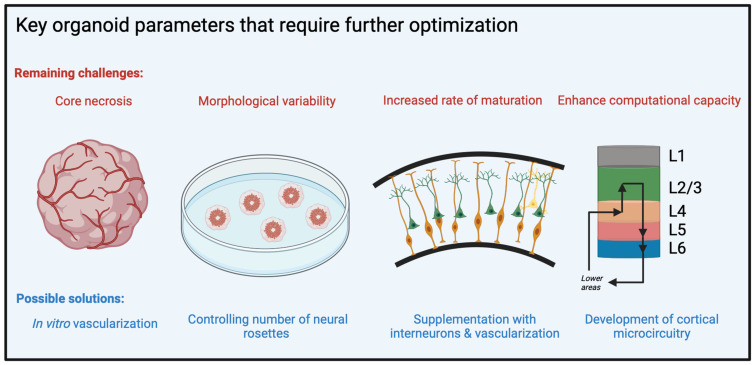
**Obstacles to the use of organoids in transplantation-based cortical repair.** Brain organoids currently lack vascularization, leading to internal necrosis due to lack of sufficient oxygen perfusion and nutrient supply. Organoid culture protocols produce morphologically heterogeneous organoids which are highly variable in size and number of neural progenitor zones. An optimal organoid age for specific cortical repair applications must be more precisely identified. A stronger understanding of organoid microcircuitry, such as the presence of a cortical microcircuit, is important for improving the utility of organoids in neural repair.

In the remainder of this section, we discuss recent progress in the organoid field relevant to their suitability as substrates for use in neural transplantation, including cell type diversity and potential for regional specification, cortical architecture, electrophysiological function, and graft survival and appropriate integration. In addition, we consider remaining challenges to the clinical implementation of organoids as a repair substrate (Figure 3).

**c.**  **Cell type diversity and potential for regional specification**

Brain organoids reproduce the cell type diversity of the brain to a significant extent, containing a range of neural cell types as well as human-specific outer radial glia, astrocytes, and oligodendrocytes [111,114,115]. Brain organoids can be generated using whole-brain protocols that produce a rich diversity of brain cell types [116,117]. However, these entities demonstrate significant variability in cell populations across both organoid batches and stem cell lines [117]. Region-specific protocols represent an alternative approach, leveraging small molecules to guide differentiation. The past decade has seen tremendous progress in this area, with organoids recapitulating dorsal forebrain [118,119,120], hippocampus [121], thalamus [122], hypothalamus [111], cerebellum [123], ventral forebrain [124,125,126], midbrain [127], and choroid plexus [128]. Regional specification is meaningful in the context of neural transplantation since the regional identity of engrafted neurons is crucial to effective reconstruction of cortical circuitry [104,129,130]. In addition, region-specific organoids have been shown to be have less batch-to-batch and inter-batch variability than whole-brain organoids [131], representing a meaningful advantage for clinical translation.

While organoids derived using region-specific differentiation protocols may lack the full complement of cell types, organoids may be supplemented with underrepresented cell types such as interneurons. The Gage group recently demonstrated the feasibility of supplementing organoids with other cell types by adding microglia to cortical organoids prior to transplantation into mouse retrosplenial cortex [132]. Assembloids have also been created by fusing two different region-specific organoids, including cortico-striatal [129], cortico-thalamic [126,133], and cortico-motor assembloids [134]. This approach may similarly be used to incorporate interneurons into organoids [124,125,135], thereby enhancing their potential as a substrate for transplantation-based repair.

**d.**  **Electrophysiological activity**

Importantly, brain organoids can be maintained for the long culture times necessary for functional maturation. Cortical organoids have been shown to demonstrate steady increases in firing rate, synchronicity, population spiking events, and burst frequency during 10 months in culture [136,137]. Furthermore, whole-brain organoids have recently been shown to have action potentials with inter-spike intervals which are exponentially distributed and have Poisson-like spike trains [138]. Notably, this activity is known to be an emergent feature of primate cortex [139]. Flexible mesh multielectrode arrays have also been developed, allowing for the capture of activity from a larger number of sites on the surface of an organoid [140,141,142]. However, such approaches are limited by the inability to record from single cells or from the interior of an organoid. 

Oscillatory activity has also been observed in cortical organoids in vitro [135,143]. Notably, oscillations were only observed when cortical organoids were supplemented with interneurons [135]. Consequently, the authors of this study suggested that the emergence of multi-frequency oscillatory activity in organoids is dependent upon the presence of inhibitory interneurons. The balance between excitatory and inhibitory synaptic inputs within a neural circuit is highly important to brain function, with specific interneuron subtypes supporting electrophysiological features such as feedforward inhibition, feedback inhibition, and disinhibition [144]. Organoids, particularly region-specific, cortical organoids, are known to lack somatostatin-positive and parvalbumin-positive interneurons [145], two important populations which migrate from the ganglionic eminence to the dorsal telencephalon during development [146,147]. Oscillations have also been reported in unfused whole-brain organoids [138], which are known to contain a wider range of neural cell types albeit in stochastic proportions [117]. Overall, this suggests that a more representative number of inhibitory interneurons may allow organoids to more closely approximate normal cortical activity.

**e.**  **Graft survival and integration**

Recent organoid transplantation studies have demonstrated robust graft survival [132,148,149,150,151]. For example, Revah et al. and Jgamadze et al. similarly observed surviving organoid grafts in >80% of transplanted animals at 2 months and 1, 2, and 3 months post-transplantation, respectively. By comparison, attempts at transplantation of dissociated neurons have observed <10% survival of engrafted cells [152], with low graft survival also seen in other studies using injections of dissociated cells for injury repair [153,154,155]. This large difference in survival rate of structured grafts versus dissociated cells has also been noted in embryonic tissue transplants, with pieces of engrafted tissue performing better than injection of a cell suspension [156,157]. 

Remarkably, brain organoid grafts achieve functional integration with host circuits. They show brain region-specific electrophysiological responses, as demonstrated using visual stimulation [158,159] as well as sensory stimulation [150]. Notably, neurons in engrafted organoids have also been shown to possess more sophisticated function such as orientation selectivity [148]. There is also evidence that stimulation of grafts can affect animal behavior, although not in a brain region-specific manner [150]. This degree of functional integration supports the translational potential of organoids.

**f.**  **Challenges and possible next steps**
  - **Vascularization**

Current organoids lack vascularization, leading to inadequate nutrient supply, oxygen perfusion, and exchange of waste. These factors result in the development of necrosis at the core of the organoid as it enlarges, limiting the size to which healthy organoids can be grown. While several approaches have been used to overcome this constraint, including organoid slicing [113], agitation of media [116], use of air-liquid interfaces [158], and use of hyperoxic conditions [118], further optimization work remains necessary. 

Several strategies for brain organoid vascularization may be viable to pursue. The co-culture of stem cells with human umbilical vein endothelial cells (HUVEC) prior to neural differentiation has been used to generate pre-vascularized cortical organoids [160]. This approach has the advantage of generating organoids with fully integrated blood vessels as early as day 42 in vitro, likely before any significant necrosis may occur. However, the consequences of HUVEC signaling during neural differentiation requires further exploration, as it is possible that the presence of HUVECs may perturb neural patterning. Another approach involves using doxycycline-inducible ectopic expression of the embryonic transcription factor human ETS variant 2 to begin vascularization in day 18 organoids [161]. A third protocol relies on the application of soluble factors such as vascular endothelial growth factor and angiopoietins to stimulate vascular growth in partially differentiated organoids [159]. These latter two strategies fail to generate branched vascular networks, instead producing a small number of large but disconnected vessels throughout the organoid. Furthermore, in all of these methods of organoid vascularization, the blood vessels still lack a central pump to create a fully functioning circulatory system. A microfluidic approach to vascularization, as demonstrated by the Huh and Ingber groups in placental and lung organoids, respectively [162,163,164], may enable dynamic fluid flow through organoids. Notably, organoid vascularization is required for development of a blood-brain barrier, which plays a key role in regulation of substances entering the brain [165]. However, it is unlikely that organoid vascularization alone is sufficient for establishment of a true blood-brain barrier, given the absence of important factors such as shear stress provided by fluid flow, as well as a full physiological complement of pericytes, oligodendrocytes, and immune cells [159,160,161,165]. Overall, further refinement may be required to develop robust strategies for brain organoid vascularization.

ii. **Variability in culture**

In addition to cell type diversity, it is important to consider morphological variability in organoids. Currently, cortical organoids have high variability in their number of cortical units. These rosettes, comprised of Pax6^+^ neural progenitor cells surrounded by concentric layers of neurons, resemble the human ventricular and subventricular zones. The formation of multiple neural rosettes within organoids represents a challenge for restoration of cortical structure and translation. Several approaches to producing single-rosette organoids have recently been studied. A monolayer culture of neuroepithelium may be differentiated within a micropatterned array, which geometrically constrains growth to promote formation of a single neural rosette [166]. Rosettes are then released from the array and maintained in suspension culture. In an alternative method, rosettes are manually cut out of monolayer cultures to form organoids, which are subsequently transferred to suspension culture [167,168]. These additional processes increase the cost of organoid generation and decrease overall yield. However, increasing the reproducibility of organoid size and rosette structure will ultimately be important for clinical translation.

iii. **Timeline of maturation**

Brain organoid maturity is a parameter that requires careful consideration in the context of transplantation outcomes. Cortical organoids have been shown to mature at a similar rate to neurons in the developing human brain [117,131,169], with transcriptomic and morphological features of day 250–300 organoids generally reflecting those of postnatal brain [169]. Furthermore, hPSC-derived organoids have been shown to follow a specifically human developmental timescale, distinctly longer than even those of organoids derived from cell lines obtained from nonhuman primates [170]. It may be possible that less mature organoids are optimal for certain cortical repair applications, while more mature organoids are better suited for others. Consequently, further research is necessary to identify the ideal organoid age for use in specific cortical repair procedures. Once an ideal age of organoids is determined, efficient organoid transplantation and integration may require manipulating the maturation of organoids. 

Several strategies have been proposed to accelerate organoid maturation in vitro. Organoid vascularization has been demonstrated using several methods, as discussed above. Such approaches aim to generate organoids containing blood vessels, thereby preventing internal necrosis and more closely approximating normal brain. These studies have reported an increase in mature neurons within organoids, despite the majority lacking fluid flow through their vascular networks. Supplementation of organoids with microglia, a cell type which is absent in current brain organoids, has also been demonstrated to increase neural maturation [171,172]. Microglia-supplemented organoids were shown to have increased cell maturity based on single-cell transcriptomic analyses as well as more mature electrophysiological properties in multi-electrode array recordings. Notably, this result suggests that supplementation of organoids with other underrepresented cell types, such as inhibitory interneurons, may represent a promising means of accelerating maturation.

iv. **Cortical circuitry**

Obtaining a stronger understanding of organoid neural circuity is important for better defining the utility of organoids as a substrate for neural repair. However, the development of cortical microcircuitry which is characteristic of neocortex, such as the canonical cortical microcircuit described earlier, has not been more explicitly studied in organoids. The presence of organized microcircuitry in neural grafts may enable greater restoration of function in the injured brain. While extensive cellular characterization of organoids has been performed, organoid microcircuitry has not yet been explicitly mapped. Therefore, the development of organoid microcircuitry represents an important avenue for future study of structured grafts. 

## 5. Conclusions

Global collaboration has led to significant progress in developing neural repair therapies, with more widespread clinical translation appearing increasingly likely. As the field of central nervous system cell replacement continues to advance, it is prudent to consider that neural replacement therapies should be guided by the specific design needs associated with the neurological condition being treated and its relevant neuroanatomy and neural circuitry. The high structural and functional complexity of the cerebral cortex means that it is improbable that long-term treatment of cortical injury will be achieved through injections of dissociated cells. Rather, grafts will likely need to be highly structured to enable efficient structural and functional integration with host brain. By comparison, the distinct pathology of PD—extensive loss of midbrain DA cells—supports the use of injections of dissociated cells in the striatum. While unstructured grafts of DA progenitor cells may provide therapeutic benefit for PD by restoring dopamine secretion, subsequent research may achieve even greater symptom relief and avoidance of side effects through strategies based on reconstruction of the circuitry of the basal ganglia.

The future of neural replacement therapy will likely involve the use of a wide range of specific neuronal subtypes, immunomodulatory strategies, and advanced surgical techniques to enable more extensive circuit reconstruction. These may target a multitude of neurological conditions, with individual therapeutic approaches tailored to address specific disease pathophysiology and corresponding design needs. The integration of immune cells into neural grafts has shown promise in preclinical models of Parkinson’s disease [60] and, more recently, of Alzheimer’s disease [173]. For example, the restoration of microglial function represents a plausible approach to treatment of Alzheimer’s-associated neurodegeneration [173,174]. Immunomodulation may continue to be a significant focus in tissue repair, encompassing strategies such as immune checkpoint blockade [175,176] and immune cell engineering [177]. Specifically in the realm of cortical repair, there will likely be a need to optimize the neuronal subtype composition of grafts to match the areal identity of damaged tissue. Additionally, the computational capacity of grafts may be enhanced to support the function of native brain, perhaps through supplementation of grafts with key interneuron populations. The excitatory-inhibitory balance within a neuronal network is essential to network activity and has been shown to be dysregulated in conditions such as Alzheimer’s disease [178]. In addition, strategies such as pre-transplantation graft entrainment using optogenetic and chemogenetic tools may increase functional activity in cell grafts. The integration of structured grafts with existing circuits may also be further optimized through the use of small molecules and graft-pre-vascularization. Alternatively, gene therapy may be used for direct neuronal reprogramming from glia to achieve neural replacement in conditions such as stroke [179]. For example, the neurogenic capacity of subsets of astrocytes may enable the use of local astrocytic cells for limited replacement of neurons in the setting of more limited injury [180,181]. In addition, there is evidence that ependymal cells may become neurogenic following stroke-like injury, with inhibition of Notch signaling promoting a proliferative response [182]. Finally, neural replacement strategies may be informed by gene expression and multi-regional analyses to develop a stronger understanding of disease-based design needs [183]. Collectively, the combination of methods to generate neuronal subtypes to reflect those lost in a given disease, advances in transplantation techniques, and novel genetic tools may yield more powerful and personalized therapies. 

## Figures and Tables

**Table 1:** Ongoing clinical trials of dopamine cell replacement for treatment of Parkinson’s.

Cell Source		Title	Sponsor	Phase	Number of Participants	Immuno-Suppression Used	Trial Start	Clinical Trial ID	Reference
hESC/NSC-derived DA progenitors	Parthenogenetic hNSCs	A single arm, open-label phase 1 study to evaluate the safety and tolerability of ISC-hpNSC injected into the striatum and substantia nigra of patients with Parkinson’s disease	Cyto Therapeutics	I	12	Yes	2016	NCT02452723	Garitaonandia et al., 2016 [85]
	hESC-derived mDAPs (MSK-DA01)	Phase 1 safety and tolerability study of MSK-DA01 cell therapy for advanced Parkinson’s disease	BlueRock Therapeutics/Memorial Sloan Kettering/Weill Cornell	I	12	Yes	2021	NCT04802733	Piao et al., 2021 [20]
	hESC-derived mDAPs (STEM-PD)	STEM-PD trial: A multicentre, single arm, first in human, dose-escalation trial, investigating the safety and tolerability of intraputamenal transplantation of human embryonic stem cell derived dopaminergic cells for Parkinson’s disease (STEM-PD product)	Lund University/Cambridge University	I/II	8	Yes	2022	EudraCT-2021-001366-38	Kirkeby et al., 2023 [86]
hiPSC-derived DA progenitors	Autologous hiPSC-derived mDAPs	Transplantation of autologous midbrain dopaminergic neuron precursors derived from a Parkinson’s disease patient’s induced pluripotent stem cells	Harvard University	N/A	1	No	2017	IND17145	Schweitzer et al., 2020 [83]
	Allogeneic hiPSC-derived mDAPs	Kyoto trial to evaluate the safety and efficacy of iPSC-derived dopaminergic progenitors in the treatment of Parkinson’s disease	Kyoto University Hospital	I/II	7	Yes	2018	UMIN000033564	Takahashi, 2020 [82]
	Autologous hiPSC-derived mDAPs (ANPD001)	To be announced	Aspen Neuroscience	I/IIa	Recently cleared for enrollment	No (*planned*)	To be announced	To be announced	
Aborted human fetal mesencephalic tissue	Fetal ventral mesencephalon (TRANSNEURO)	An open label study to assess the safety and efficacy of neural allo-transplantation with fetal ventral mesencephalic tissue in patients with Parkinson’s disease	University of Cambridge	I	11	Yes	2012	NCT01898390	Barker and TRANSNEURO consortium, 2019 [87]

## Data Availability

No new data were created or analyzed in this study. Data sharing is not applicable to this article.
